# Quality assessment in primary health care: Adolescent and Youth Friendly Service, a Mozambican case study

**DOI:** 10.11604/pamj.2020.37.1.22983

**Published:** 2020-09-01

**Authors:** Emiliana Bomfim, Martins Abudo Mupueleque, Delmar Mário Mutereda dos Santos, Ahmed Abdirazak, Regina de Arminda Bernardo, David Zakus, Paulo Henrique das Neves Martins Pires, Ron Siemens, Celso Fernando Belo

**Affiliations:** 1 College of Medicine, University of Saskatchewan, Saskatoon, Canada,; 2 Mussa Bin Bique University, Nampula, Mozambique,; 3 Marrere Health Centre and Hospital Director, Nampula, Mozambique,; 4 Faculty of Health Sciences, Lúrio University, Nampula, Mozambique,; 5 Dalla Lana School of Public Health, University of Toronto, Toronto, Canada,; 6 Pediatric Emergency Medicine at the University of Saskatchewan. Canadian Co-PI of the Alert Community Prepared Hospital Project, Saskatoon, Canada

**Keywords:** Adolescents, family planning, Mozambique, primary health care, sexual and reproductive health, youth

## Abstract

**Introduction:**

despite the Mozambican Ministry of Health's efforts to deliver family planning to all girls of childbearing age, the adolescent pregnancy rate remains high. The Adolescent and Youth Friendly Service (AYFS), integrated into overall primary health care programs throughout the country, aims to reverse this situation. Our study objective was to assess this health care service's quality in its location in Marrere Health Centre, Nampula, northern Mozambique, using clients' perspective.

**Methods:**

we implemented a descriptive cross-sectional quantitative study sampling 124 individuals, who had recently accessed the AYFS at Marrere Health Centre. Data were collected through a questionnaire using a 5-point Likert scale in questions regarding satisfaction level (i.e. always, most times, sometimes, few times, never) and additional open answer questions to gain greater specific understanding.

**Results:**

a total of 126 users of the AYFS were evaluated, all from the Emacua ethnic-linguistic group. 85 (67%) were adolescents (<19 years), 78.2% female. The mean age was 17.6 years. We found an average of 0.54 pregnancies per woman and 87 participants (69%) never had a pregnancy; of 39 (31%) who had been pregnant, 17 (44%) were able to report the date of the first prenatal visit, on average performed at week 16 (2nd trimester), though with 9 (53%) having performed it during the first trimester. Spontaneous and induced abortions were reported respectively in 4 and 34 cases, respectively, and none with adolescents. The “overall satisfaction” rate was more frequent in both groups, being answered by 93.8% of youth and adults (>= 19 years) and 72.0% of adolescents, a statistically significant difference between the two groups (p <0.05).

**Conclusion:**

while most users are satisfied with the services there was, however, some sharp criticism. Health professionals' practice with the protocol varied, and there were significant deficiencies in information and communication with users. Open communication within families and information reinforcement about sexual and reproductive health and male participation in family planning were found to be in need of strengthening. Our recommendations include reinforcing health professional's training to protect adolescents and young people' sexual health, an important strategy in primary health care to achieve universal health coverage.

## Introduction

Adolescent pregnancy is one of the causes of Mozambique’s high maternal mortality rate (451.6 maternal deaths per 100,000 live births in 2017) [1-3], with almost half of girls aged from 15 to 19 years having one child or had been pregnant. These teenage mothers are more likely to suffer serious complications and death as well as new-born problems and morbidity [4]. The highest mortality ratio is in the 12-14 years age group (1,816 maternal deaths per 100,000 live births). Approximately 20% of maternal deaths occur in girls who have not reached their twentieth year and 14% as a result of abortion [5]. Pregnant adolescents face a higher risk of maternal mortality, as they are not sufficiently physically developed for birth, and can potentially suffer health problems such as obstetric fistula and unsafe abortion. When a girl becomes pregnant, her present and future change radically. The likelihood of dropping out school increases, employment opportunities diminish, their health is at risk, family problems can ensue and their vulnerability to poverty, stigma, exclusion and dependency worsens [6]. At 14 years of age, 33% of Mozambican adolescents become sexually active and their knowledge about human immunodeficiency virus (HIV) infection prevention is weak and girls are particularly vulnerable, being three times more likely to be HIV infected than boys. Women between 20 and 24 years have a fourfold higher chance to be infected than men and adolescents between 15 and 19 a threefold probability. Most boys between 15 and 19 know at least one HIV prevention method, much more than girls. Usually, adolescent girls do not have the power to refuse unprotected sex and only 12% of girls aged 15-24 reported using condoms on their last sexual intercourse [7]. Community home-based studies have shown that 37.5% of adolescents get pregnant for the first time between 15 and 19 years of age [8]. The Mozambican Demographic and Health Survey in 2011 showed that among men and women between 15 and 24 years that never married, 67% of men and 44% of women that had sexual intercourse in the last 12 months, more than half did not use condoms.

This is the reality for many adolescents and young people, mainly those living in rural areas, with lower school levels belonging to families with scarce economic resources. The consequences in Nampula province are girls between 15 and 18 years of age frequently dropping out of school and alleging sexual abuse without protection, resulting in pregnancy. This issue compromises the Mozambican Government´s plan to improve girls´ education. The Mozambican Government has implemented several interventions in the health sector to protect and promote adolescents´ development. Information and education campaigns have been a way to approach adolescents´ issues [9]. Government and Non-governmental Organisations (NGOs) activities have increased human rights knowledge and reduced domestic violence but have had a low impact on girls´ education. Since 1998 there has been a program, the Adolescent and Youth Friendly Service (AYFS), integrated within the National Health Service (NHS), providing health care services, sexual and reproductive health (SRH) education and counselling, ultimately to empower them with life skills. The service is free, confidential and provided by health professionals (HP) trained to deal with young people, provided as part of primary health care (PHC) services in all health centres (HC). Services offered include education and information on contraceptive methods, emergency contraception, STI prevention and treatment, antenatal care, and postpartum or post abortion counselling, as well as condoms and HIV information. In these services, peer educators hold lectures, promote debates and other educational activities.

Those facts led the Lúrio University (UniLúrio) Faculty of Health Sciences (FHS) and the University of Saskatchewan in Canada, as part of the Alert Community to Prepared Hospital care continuum (ACPH) implementation research project (aiming to reduce maternal and neonatal mortality rates at the Natikiri Administrative Post, Nampula municipality), to assess the quality of care at Adolescent and Youth Friendly Service (AYFS) in Marrere Health Centre (MHC) - the referral primary care health unit (HU) in the area. The study took place during the 5th semester of the project (June 2019), which had been in progress already for two years to educate about SRH and family planning (FP) and to encourage the population to participate, training HP in humanized, patient centered family friendly care, as well as introducing technological improvements into MHC. The project´s baseline study had shown the population's low level of knowledge on SRH and FP [10]. Thus, we intended to evaluate AYFS user´s opinion on the quality of care and service in MHC, as well as to assess if there were any differences between children and adolescents in the “overall satisfaction” rate.

## Methods

We implemented a cross-sectional quantitative descriptive study, focused on users of the AYFS consultations at MHC. The sample size was drawn, considering an 8% error margin, from the service users´ total population (708 users) during the first semester of 2019. The desired level of accuracy for each indicator was based on a 95% confidence level. To calculate sample size we used:

$$n=\frac{N*Z^{2}*P*\left(1-P\right)}{Z^{2}*P*1-\left(P\right)+E^{2}*N-1}$$

(n = sample size, N = size of the study population, Z = confidence level (0.95), p = constant 0.5, E = margin of error (0.05). The number calculated for the sample was 124 and the subjects were subsequently selected by convenience sampling and sampled consecutively until sample size was achieved. Data were collected with a survey with questions regarding satisfaction level using a 5-point Likert scale (i.e., always, most often, sometimes, few times, never), along with some open answer questions. Survey´s main outcomes were evaluation of good care and service principles, communication with the patient, privacy and confidentiality, information about FP and general satisfaction. Open-ended questions explored more deeply the strengths and weaknesses of service delivery. The survey was pretested and applied by students from the UniLúrio FHS Health Research Students Board, duly trained, unknown to the subjects and after signing an ethical and scientific commitment term, from June 28 to July 3, 2019. The study included participants available at AYFS visits who voluntarily agreed to participate. All respondents signed an informed consent term, including an informed consent term for adolescents under 19 years of age. Assent from the children and consent and permission from the parents, guardians, or other legally authorized representatives, were obtained when both agreed to participate in the study. Participants were surveyed and responded verbally in *Emacua* (local language) or Portuguese, as they wished, to the inquirers' questions for about 20 minutes.

Survey data were typed into a secure database created and hosted by the University of Lurio. Data were uploaded to this database by students supervised by a statistics professor, then analyzed with Statistical Package for Social Sciences (SPSS) 22.0 software. Users were considered satisfied when, for each question, they answered “always” and “most of the time” and were “satisfied” and “very satisfied”; their frequencies, means and standard deviation were calculated. Study´s variables were age in years, gender, place of residence, school level, number of pregnancies, place of delivery, number of abortions and date of first prenatal visit. A Student´s t-test was performed to assess if there were any differences between adults and adolescents in the “overall satisfaction” rate. This research met all Helsinki Declaration (2013) recommendations and was approved by the Institutional Committee on Health Bioethics at UniLúrio, Nampula (02/CBISUL/16), as well as by the Behavioural Research Ethics Board of the University of Saskatchewan, Canada (protocol #: 15-112).

## Results

A total of 126 AYFS users were evaluated, all from the *Emacua* ethnic-linguistic group, including 85 adolescents (<19 years) and 97 (78.2%) female users, of which 69 (82%) were adolescents. The average age was 17.6 years (range 12-38) and for adolescents 15.6 years (range 12-18). The level of school education is detailed in Table 1. To validate our findings, the monthly statistical summary of the service, which records users´ characteristics and the type of services provided at AYFS, was reviewed. The educational level of our study participants was confirmed by the medical records. As stated in Table 2, most users were adolescents and female, with completed primary education, validating the findings of our study. Of the referred 122 households, 108 (89%) were living in Natikiri neighborhood, while the others were from surrounding areas, but outside of Natikiri catchment area. Regarding the number of pregnancies, we found an average of 0.54 pregnancies per adult woman (range 0-8), and an average of 0.09 for adolescents (range 0-3). Of 126 users, 87 (69%) had never had a pregnancy; of the 39 (31%) who had been pregnant, 22 did not know the date of the first prenatal visit, which 17 (44%) were able to report. On average, 9 (53%) attended prenatal visits during the first trimester and 40% attended during the second trimester. Regarding the number of hospital deliveries, we found an average of 0.46 hospital deliveries per user (range 0-6), with 0.05 hospital deliveries per adolescent (range 0-3). Only five home births (range 0-2) were reported. Spontaneous and induced abortions were reported respectively by 4 and 34 adult women (average of 0.03 per user), none of these spontaneous abortions were reported by adolescents. A total of 34 cases of induced abortion were reported (an average of 0.27 per user), with none of these induced terminations being reported by adolescents. Principles of good care evaluation are detailed in Annex 1. We highlight the following results: the HP asked for client´s name (always: 81%); users were greeted and offered a seat (always: 72%); users felt welcome to the consultation (always: 62%); are motivated to return to the consultation (always: 60%, mostly: 17%). Other answers are less favorable: the HP did not present her/himself (never: 58%); a small majority explained what would happen in the consultation (always: 41%, most times: 13%), clarifies questions related to health status (always: 38%), asks for questions at the end of consultation (never: 51%); or summarizes most important information at the end of the consultation (always: 39%, most of the time: 6%).

**Table 1:** Adolescent and Youth Friendly Service visit users' school education level

Participants	Total n=124		Adolescents n=84	
School education level	N	%	N	%
Illiterate and incomplete primary school	14	11	9	11
Complete primary school	100	81	72	86
Complete secondary school	10	8	3	3
Higher education or techno-professional	0	0	0	0
Total	124	100	84	100

**Table 2:** Adolescent and Youth Friendly Service medical records on monthly visits statistics, August 2019

nº	Type of service	Age group (years)		
		10-14	15-19	20-24
	
1	1^st^ appointment Female (n)	27	123	88
2	1^st^ appointment Male (n)	21	32	40
				
		**All age groups**		
3	Total 1^st^ consultations (n)	331		
4	Schooled (n)	218		
5	Unschooled (n)	113		
6	% schooled	65,9		
7	HIV screening (n)	81		
8	HIV + test (n)	3		
9	HIV incidence (%)	3,7		
10	No. of pregnant women (n)	17		
11	Syphilis Screening (n)	118		
12	Syphilis Test + (n)	3		
13	Syphilis incidence (%)	2,5		
14	1^st^ contraception (n)	31		
15	1^st^ contraception/1^st^ consultation (%)	9,4		
16	2nd contraception (n)	26		

Caption: n - number of patients; HIV - Human Immunodeficiency Virus; % - percentage

Regarding communication with the user, the HP encouraged the participation of the husband or partner (never: 66%), clarified doubts in friendly manner using simple and clear language (never: 30%); and encouraged little to ask questions (never: 46%). Asking about user expectations never occurred with 58% and explanation of the physical examination was mentioned only by a small majority (never: 45%). Visit privacy was mentioned by the majority of users (always: 52%, most times: 12%) and the absence of strangers or unsolicited health professionals in the room (always: 57%, most times: 12%) was better guaranteed. Confidentiality of information was stated as never by 50% as was consent to talk to other family members about the prenatal visits (never: 48%). Less than half of users were informed about the benefits of FP (never: 58%) and a little more about three different types of contraceptives (never: 46%); only a small minority was informed about their side effects (never: 71%). In the overall assessment of user satisfaction of the AYFS visits, the majority is satisfied (Table 3). The “overall satisfaction” rate was more frequent in both groups, being answered by 93.8% of adults, and 72% of adolescents, a statistically significant difference between these groups (p<0.05).

**Table 3:** Adolescent and Youth Friendly Service visits users' satisfaction level

Participants	Total		Adolescents	
Satisfaction level	N	%	N	%
Very satisfied	26	21	20	24
Satisfied	64	52	40	48
Unhappy	27	22	18	21
Dissatisfied	7	5	6	7
Total	124	100	84	100

Answers to the open-ended questions included: 1) What did you like more about the service? A total of 108 users (87%) liked it, and highlighted the following: good service, promptness, information, professional empathy, availability of medication, contraceptives and blood tests, counselling; a total of 4 (3%) did not comment; and 12 (10%) did not like it. 2) What issues you did not like in the service? A total of 72 users (58%) liked everything, not mentioning any complains; 5 (4%) did not comment and 47 (38%) did not like it, highlighting the following deficiencies: delay in care, no clarification of doubts, uncomfortable chairs, lack of respect and aggressiveness of the HP. 3) What would you change to have better care? A total of 64 users (52%) said nothing; 10 (8%) did not answer; and 50 (40%) had several suggestions, such as improving HP quality of care (14%), HP communication skills (9%), respect the order of patients´ arrival (2%), faster attendance (2%), improve hygiene (2%), avoid the use of cell phone during consultation by HP, improve privacy (2%), avoid visits and interruptions during consultation (2%) and remove low performing HP (each 2% respectively).

## Discussion

We surveyed a group of mostly adolescent and female users, with completed primary education. Our findings show that most users were satisfied with AYFS consultations, but adolescents generally report slightly lower quality care, except in the areas of privacy and contraceptive information, which may reveal a lack of HP specific preparation to care for this age group. Other studies have reached the same conclusions on satisfaction of users regarding prenatal care in low income countries [11,12]. Interestingly, many studies that found a high level of overall satisfaction with prenatal care also find important deficiencies in other aspects related to antenatal care, for instance, satisfaction about information on labor, delivery, FP, pregnancy complications, visit spacing and private issues. Therefore, previous studies have questioned the validity of this high level of satisfaction associated with a high number of deficiencies in the antenatal care service. It is unknown if a high satisfaction regarding antenatal care could be an extent of passivity or intimidation, which varies according to user´s social class and the amount of psychosocial support they receive throughout pregnancy [13].

Many studies have investigated why women do not use antenatal services in low- and middle-income countries, and previous research with both youth and adolescents have concluded that adolescents' perceptions about the care they receive can have a decisive influence on their decision to seek SRH services. If they fell that they are poorly treated by HP, stigmatized or ashamed to be sexually active, they will be discouraged to turn to these health services [14]. Sexual and reproductive rights (SRR) are rights that all people, regardless of age, gender and other characteristics, have, to make choices regarding their sexuality and reproduction, while respecting the rights of others. This includes the right to access information and support services for these choices and the promotion of SRH. Investing in individuals in their early teens is crucial. Generally, girls are more likely to engage in early adolescent sex but are less likely to use contraceptives, due to gender and empowerment issues, in which men have the power over condom use and girls have little negotiation power. A frequent consequence is pregnancy, which promotes forced premature marriage, which often too has poor outcomes, such as loss of educational opportunities. Teenage pregnancy frequency is directly linked to premature marriage, as 18% of girls in this age group are married before the age of 15, despite federal laws prohibiting premature marriage. In Nampula Province, 52% of women get married before age 18, in rural areas 56%; of these, 21% get married before the age of 15. This scenario places Mozambique as one of the seven countries in the world with the highest number of premature marriages. AYFS users live predominantly in rural areas, where parent-child communication about SRH faces many cultural barriers; they have a low level of school education and about one third already had a pregnancy; of those, more than half were unaware of the date of the first prenatal visit; half of those who knew this date did it in the first trimester, which is in line with other studies in developing countries [15].

A previous study has shown that empowering adolescents to exercise their reproductive rights is a challenging exercise. In Mozambique, teenage pregnancy rates are influenced by the level of education: with 1 in 2 adolescents who have not received formal education becoming pregnant to 1 in 4 among girls with secondary or higher education. Additionally, adolescent fertility rate reached 167 births per 1,000 girls (2011) and 42% of women aged 20-24 had their first child before turning 18. Our study found that most respondents were not informed about all types of available contraceptive methods or about their side effects. The high adolescent fertility rate may be associated with several aspects, which can include one or more of the following: poor knowledge about FP options and methods to avoid pregnancy; poor knowledge about even the possibility of FP; and a lack of knowledge about pregnancy in general. We also noted that HP do not generally proceed according to humanized care standards and the Ministry of Health (MISAU) protocol, presenting deficiencies in communicating with users (e.g., lack of opportunities to address patients´ questions, poor information on contraceptives). Additionally, most participants indicated that HPs did not request consent before talking to their partners or family and at the end of the visit did not ensure that their personal information would not be discussed with others. Interventions to reduce unmet contraceptive needs and unwanted early pregnancies in adolescents are very important components of FP programs, especially in developing countries like Mozambique [16]. Achieving Mozambique's Sustainable Development Goals for adolescent health will require increased investment in the AYFS program with reliable and up-to-date indicators on coverage, geographical inequality and good quality of care [17,18]. Therefore, programs such as AYFS aiming to provide information and contraceptive services to adolescents and youth, is an important element in guaranteeing human rights, as well as in achieving national health goals and universal health coverage [19].

Important limitations of this study must be noted. First, we point out the location where the surveys were applied (i.e. waiting room), where some responses may have been influenced by the environment. Waiting room surveys can generate unique material for evidence-based primary health care practice, however, a limitation arising from this type of method, is that, even though consent is sought from all participants, some might not be comfortable with interrogation, and/or feel intimidated. In order to mitigate this problem, staff training (i.e. interviewers) was performed, as well as interviewers were selected for having excellent people skills and for being fluent in the local language. A second limitation of this study was the application of the Likert rating scale, particularly to a population with difficulty of abstract conceptualization and due to limitations in the translation into local language of certain terms, such as “always”, “often”, “sometimes and “few times”, which, therefore, might have made it confusing to understand. To counteract this limitation, interviewers were encouraged to spend enough time giving different examples to illustrate the meaning of these terms in the local language. Future studies should consider using emoji to complement the traditional numerical Likert scale.

## Conclusion

Most AYFS users are satisfied with the services, however, with some sharp criticism, which can be used to improve services. HP practice is poor with partial adherence to service protocols, but there are significant deficiencies in the type of information and manner of communication with adolescent users, especially regarding FP. It is recommended to strengthen the supervision and preparation of AYFS professionals (including continuing education programs), aiming to better promote and disseminate FP in PHC and the protection of adolescents and young people´ health and social position in a more effective and respectful way. Open communication within families and information reinforcement about SRH and male participation in FP also need to be strengthened. In addition, it is recommended to involve stakeholders in a broader and multilevel approach to provide sexual and reproductive health (i.e. schools, church, teachers, traditional healers etc.). The AYFS is an important strategy to achieve national goals and universal health coverage and enable a safer and more secure future for adolescents and youth.

### What is known about this topic

- In many developing countries like Mozambique, the adolescent pregnancy rate remains high, along with a low access and the utilization of FP;
- Many are the barriers to the use of FP by adolescents and youth. Although some of these barriers exists at a patient level, there is a scarcity of studies trying to understand the level of satisfaction of this population;
- Most of the existing evidence on the satisfaction of users towards sexual and reproductive health services are general and not tailored to a specific Adolescent and Youth Friendly Service, therefore, their findings vary across populations.

### What this study adds

- Provides information on quality of care and user's satisfaction on Adolescent and Youth Friendly Services. Our results show that the “overall satisfaction” rate was high in both age groups, with a statistically significant difference;
- Although most users are satisfied with the services, some sharp criticism were incorporated into analysis. Health professionals' practice is poor with partial adherence to service protocols, added to significant deficiencies in information and communication with users.

## Annex

**Annex 1**: users' opinion responses about the quality of Adolescent and Youth Friendly Service (AYFS) in Marrere Health Centre (PDF-322 KB)
